# 

**Published:** 1890-08

**Authors:** 


### Communications.

### MISSOURI STATE DENTAL ASSOCIATION.

St. Louis, Mo., July 29, 1890.

*Editor of the American Journal of Dental Science:*

Twenty-sixth annual meeting held at Pertle Springs, Warrensburg, Mo., July 8, 9, 10, 11, 1890. President Dr. Henry Fisher called the meeting to order.

The committee on resolutions to the memory of Dr. A. Noland, presented the following, which were adopted by the Association:

*To the Members of the Missouri State Dental Association:*

*Whereas*, Death has removed from our ranks our beloved brother and co-laborer, Dr. A. Noland, of Monroe City, on the 22nd of January, 1890; and

*Whereas*, Dr. Noland was a faithful student, an honored laborer, and worked hard to raise the standard of our profession in this State;

*Therefore, Resolved*, That this association mourn with sorrow the loss sustained.

*Resolved*, That this association hereby tender his bereaved family its heartfelt sympathies and condolence in this their sad bereavement, and may that God, in whom he so implicitly trusted, speak peace to their sad hearts in their distress.

*Resolved*, That a copy of these resolutions be sent to the family of our deceased brother and to the dental journals for publication.

B. Q. Stevens,
G. M. Risley,
Jas. L. Leavel,
*Committee.*

The committee to draft resolutions on the death of Dr. Judd presented the following, which were adopted :

*Whereas*, The recent death of Dr. Homer Judd brings to mind his activity and influence in the organization of this association, and his subsequent labor in extending its usefulness, to the great benefit of the profession in this State; and,

*Whereas*, His noble character, energy and his literary attainments entitle him to an exalted place on the scroll of deceased members; therefore,

*Resolved*, That in the death of Dr. Judd this Association has lost an honored member, whose professional character and example we emulate, and whose memory we ever hold dear.

*Resolved*, That the heartfelt sympathy and condolence of this Association is tendered the family of our departed brother in their sad bereavement.

*Resolved*, That a copy of these resolutions be sent to the family and to the dental journals for publication.

W. H. EAMES,
C. W. SPALDING,
J. C. GOODRICH,

Pertle Springs, July 9, 1890. *Committe.*

Drs. Price, T. W. Reed, and F. Swap were appointed a committee to draft suitable farewell resolutions to Dr. Spalding, and reported as follows :

*Mr. President and Members of the Missouri State Dental Association*,

*Gentlemen* :—We, your committee appointed to draft resolutions expressive of the pleasure of this Association, afforded by the presence of Dr. C. W. Spalding, and of regret that he will soon sever his social connections with us, most respectfully submit the following resolutions :

*Resolved*, That with a full appreciation of Dr. Spalding's virtues as a gentleman, his high moral character as a man, his eminent qualifications and invaluable counsel as a professional brother, we tender to him our most sincere thanks for his presence at this meeting (probably the last time we shall meet him on this earth); we deeply regret that he should feel it necessary to sever his social connection with us.

*Resolved*, That we fully realize the fact that no one has done more than he to advance the standard of our profession and the best interests of this Association, which will ever, by us, be remembered and cherished with grateful hearts.

*Resolved*, That in his departure, he takes with him our most earnest wishes for his success, prosperity and happiness; and may kind Providence ever watch over, guide and shield him.

*Resolved*; That these resolutions be spread upon the records of this Association, a copy, properly engrossed, be presented to Dr. Spalding, and one sent to each of the dental journals for publication.

JAS. A. PRICE,
T. W. REED,
F. SWAP,
*Committee.*

The election of officers resulted as follows: President, Dr. J. F. McWilliams, Mexico; Vice-President, Dr. Geo. L. Shephard, Sedalia; Second Vice-President, Dr. W. H. Buckley, Liberty; Recording Secretary, Dr. John G. Harper; Corresponding Secretary, Dr. William Conrad; Treasurer, Dr. James A. Price, Weston; Board of Censors—Drs. J. G. Hollingworth, W. L. Reed, Chas. L. Hungerford; Committee on Ethics—Drs. N. H. Gaines, C. V. Huff, J. W. Aikin; Publication Committee—Drs. E. E. Shattuck, H. S. Lowry, W. E. Tucker; Law—Jas. A. Price, Weston; Committee on New Appliances—Dr. J. M. Austin, St. Joseph.

EXECUTIVE COMMITTEE.—Dr. William Conrad, Dr. Henry Fisher and Dr. J. W. Whipple, St. Louis.

SUPERVISOR OF CLINICS.—Dr. A. J. Prosser, St. Louis.

Next place of meeting, Louisiana, Mo., 1st Tuesday after July 4th 1891.

WM. CONRAD,
321 N. Grand Ave. Cor. Sec'y.

---

### NATIONAL ASSOCIATION OF DENTAL EXAMINERS.

EXCELSIOR SPRINGS, MO., Aug., 1890.

*Editor of the American Journal of Dental Science:*

The ninth annual meeting of the National Association of Dental Examiners, was held at Excelsior Springs, Mo., commencing Monday, August 4, 1890.

The following State boards were represented:

Colorado, Dr. P. T. Smith.

Illinois, Dr. C. R. E. Koch.

Iowa, Drs. S. A. Garber, E. E. Hughes, and E. D. Brower.

Pennsylvania, Dr. Louis Jack.

Maryland, Dr. T. S. Waters.

Kansas, Drs. L. C. Wasson and A. M. Callaham.

Ohio, Drs. J. Taft and H. A. Smith.

Minnesota, Dr. J. H. Martidale.

During the sessions the Board of Registration in Dentistry for the State of Rhode Island and Providence Plantations, represented by Dr. Wm. P. Church, was elected to membership.

In the absence of the secretary, Dr. F. A. Levy, Dr. J. H. Martindale, of Minnesota, was elected secretary *pro tem.*

After discussion, the following resolution, offered by Dr. Jack and amended by Dr. Koch, was adopted, on motion of Dr. Taft:

*Resolved,* That this body recommends the various examining boards under no circumstances to grant temporary licenses to dental students at any period of their course of instruction, whenever their State laws will permit them so to do.

Drs. Jack, Garber and P. T. Smith were appointed a committee to formulate the principles which this association would recommend should be incorporated in the State laws. This committee subsequently presented a report which, as amended and adopted, recommended the following principles for incorporation in the laws for the regulation of dental practice or for the guidance of those framing them :

1. The creation of boards of examiners in each State.

2. The boards to be officially created by the constituted appointing power of the various State, the appointees to be selected from a number of names presented by the representative State societies; each State society at its annual meeting placing in nomination not more than two names for each appointment to be made.

3. Recognizing five years' actual practice at the time of the passage of the law as qualifying for the continuance of practice.

4. Empowering the examining boards to examine and grant certificates to non-graduates, provided the candidates present satisfactory evidence of having had at least five calendar years of instruction.

5. These and all other examinations to be both oral and written, and candidates to be also subjected to tests of practical skill.

6. Empowering the boards to examine graduates in dentistry.

7. Prohibiting medical graduates without special qualifications practicing dentistry.

8. Requiring medical graduates to have their special qualifications determined by the same tests as other non-graduates in dentistry (see No. 5).

9. Making failure to pass the required examination in any one branch sufficient cause for refusal to grant the certificate.

10. Making failure in the practical tests in either of the two general departments of dentistry work disqualification.

11. Expressing the opinion that examinations for the special degree in dentistry should be conducted by a board of examiners established by law in each State, instead of by faculties as at present; and the belief that the power to grant degrees must at length become vested in boards created for the purpose.

12. Conferring on State boards the power to revoke, for cause, a certificate of qualification previously granted.

The secretary was directed to call the attention of the American Dental Association to the fact that a case involving the constitutionality of the law regulating the practice of dentistry in New Hampshire is now pending in the Supreme Court of the United States, and asking them to see to it that it does not go by default.

Dr. Koch, from the committee on dental colleges, reported the following schools the diplomas of which this association recommends that the State boards indorse:

American College of Dental Surgery, Chicago, Ill.
Baltimore College of Dental Surgery, Baltimore, Md.
Boston Dental College, Boston, Mass.
Chicago College of Dental Surgery, Chicago, Ill.
College of Dentistry, Department of Medicine, University of Minnesota, Minneapolis, Minn.

Dental Department, Columbian University, Washington, D. C.

Dental Department of Northwestern University, Chicago, Ill. (Now University Dental College.)

Dental Department of Southern Medical College, Atlanta, Ga.

Dental Department, University of Tennessee, Nashville, Tenn.

Harvard University, Dental Department, Cambridge, Mass.

Indiana Dental College, Indianapolis Ind.

Kansas City Dental College, Kansas City, Mo.

Louisville College of Dentistry, Louisville, Ky.

Minnesota Hospital College, Dental Department, Minneapolis, Minn. (Defunct.)

Missouri Dental College, St. Louis, Mo.

New York College of Dentistry, New York, N. Y.

Ohio College of Dental Surgery, Cincinnati, O.

Pennsylvania College of Dental Surgery, Phila., Pa.

Philadelphia Dental College, Philadelphia, Pa.

School of Dentistry of Meharry Medical Department of Central Tennessee College, Nashville, Tenn.

St. Paul Medical College, Dental Department, St. Paul. Minn. (Defunct.)

University of California, Dental Department, San Francisco, Cal.

Northwestern College of Dental Surgery, Chicago, Ill. (The diplomas of this college are discredited after 1889.)

State University of Iowa, Dental Department, Iowa City, Ia.

University of Maryland, Dental Department, Baltimore, Md.

University of Michigan, Dental Department, Ann Arbor, Mich.

University of Pennsylvania, Dental Department, Philadelphia, Pa.

Vanderbilt University, Dental Department, Nashville, Tenn.

The following officers were elected for the ensuing year: C. R. E. Koch, Chicago, Ill., president; L. C. Wasson, Topeka, Kan., vice-president; J. H. Martindale, Minneapolis, Minn., secretary and treasurer. The president appointed as the committee on dental colleges, Drs. Louis

Jack, T. S. Waters, E. E. Hughes, W. P. Church, and J. H. Martindale.

On motion, the following committee was appointed to consider the advisability of holdıng the meetings at some other time and place than the annual meetings of the American Dental Association, with discretionary power in the matter: Drs. J. Taft, F. A. Levy, and S. A. Garber.

Adjourned to meet at the call of the President.

---

### NATIONAL ASSOCIATION OF DENTAL FACULTIES.

EXCELSIOR SPRINGS, MO., Aug., 1890.

*Editor of the American Journal of Dental Science:*

The seventh annual session of the National Association of Dental faculties was held at Excelsior Springs, Mo., commencing August 4. 1890.

The following colleges were represented:

Baltimore College of Dental Surgery, M. Whilldin Foster.

Boston Dental College, Wm. Barker.

Chicago College of Dental Surgery, Truman W. Brophy.

Kansas City Dental College, J. D. Patterson.

Missouri Dental College, W. H. Eames.

Ohio College of Dental Surgery, H. A. Smith.

Pennsylvania College of Dental Surgery, C. N. Peirce.

University of California, Dental Department, C. L. Goddard.

University of Iowa, Dental Department A. O. Hunt.

University of Michigan, Dental Department, J. Taft.

University of Pennsylvania, Dental Department, James Truman.

Vanderbilt University, Dental department, D. R. Stubblefield.

Louisville College of Dentistry, A. Wilkes Smith.

Indiana Dental College, J. R. Clayton.

Dental Department of Southern Medical College, L. D. Carpenter.

Dental Department of University of Tennessee, R. B. Lees.

University of Maryland Dental Department, John C. Uhler.

Columbian University, Dental Department, H. B. Noble.

On motion, Dr J. D. Patterson, Kansas City, was elected secretary *pro tem.*

The following resolution, offered by Dr. Hunt, was adopted:

RESOLVED, *That in all colleges of this association students to be graduated at the expiration of two years after admission must enter the school not later than twenty days after the opening of the regular session following this meeting.*

The amendment to the constitution laid over from last year, providing for changing the name of the association to American Association of Dental Faculties, was lost.

Applications for membership laid over last year, under the rules, were taken up and the following admitted: Royal College of Dental Surgeons of Ontario; College of Dentistry, Department of Medicine, University of Minnesota (represented by Dr. W. X. Sudduth); American college of Dental Surgery (represented by E. P. Hazen).

The following applications for membership were laid over under the rules: Dental Department of Howard University, Washington, D.C., and College of Dentistry, University of Denver.

The resolution offered by Dr. Patterson and laid over last year under the rules was taken up, amended, and adopted as follows:

*Resolved*, That after the session of 1890–91 a diploma from a reputable medical college shall entitle its holder to enter the second course in dental colleges of this association, but he may be excused from attendance upon lectures and examinations upon the following subjects: general anatomy, chemistry, physiology, and materia medica and therapeutics.

Dr. Marshall's amendment to the constitution, providing that in all matters not in conflict with Article V of the constitution a majority of the colleges belonging to this association shall constitute a quorum, was taken up and adopted.

The following resolution, offered by Dr. Hunt was adopted:

*Resolved*, That we recommend that students take two full courses in studies of a general character, such as anatomy, physiology, chemistry, general principles of surgery, and materia medica and therapeutics, and three courses in those of a special dental character.

Dr. Goddard offered the following resolution, which was adopted:

*Resolved*, That final examination may be taken at the end of the second year in three general studies.

The following, offered by Dr. Truman last year and laid over under the rules, was adopted:

*Recommended*, That for a full annual course of lectures the minimum sum of college fees be $100; that diploma fees be omitted, and an examination fee of not less than $25 be substituted therefore and made non-returnable; that a matriculation fee of $5 be charged annually. Special course fees to be $10 for each branch taken, and $5 matriculation fee.

The following officers were elected for the coming year: L. D. Carpenter, Atlanta, Ga., president; W. H. Eames, St. Louis, Mo., vice-president; J. D. Patterson,

Kansas City, Mo., secretary; H. A. Smith, Cincinnati, O., treasurer; J. Taft, Cincinnati, O., Truman W. Brophy, Chicago, and A. O. Hunt, Iowa City, Ia., executive committee.

The following committees were appointed: James Truman, Philadelphia; Frank Abbott, New York; and John S. Marshall, Chicago, *ad interim* committee; J. A. Follett, Boston; D. R. Stubblefield, Nashville, Tenn.; A. Wilkes Smith, Richmond, Ky., C. L. Goddard, San Francisco, committee on schools.

Adjourned to meet on Saturday, August 1, 1891, at 10 o'clock A. M., at the place appointed for the next meeting of the American Dental Association.

---

### Editorial, Etc.

A CHANGE OF EDITORS.—Dr. Sudduth after a very successful career as the editor of the *International Dental Jour-*

*nal*, has retired and been succeeded by Prof. James Truman, who is well known to be highly qualified for editorial work. We predict a successful era in the history of this Journal under the able management of Prof. Truman.

### To Readers and Correspondents.

Communications intended for publication, and exchange journals and papers, should be addressed to the Editor of The American Journal of Dental Science, No. 845 N. Eutaw St. Baltimore, Md. All letters relating to the business of the Journal, or containing cash remittances, to be directed to Messrs. Snowden & Cowman, No. 9 W. Fayette Street, Baltimore, Md. Articles for publication should be received by the Editor at least two weeks previous to the first of the month on which the number in which it is designed for them to appear, is issued.

☞ The American Journal of Dental Science will contain fifty pages of reading matter in each number, making a volume of about Six Hundred pages,

EXCLUSIVE OF ADVERTISEMENTS.

